# Supplementary material for: Effects of infection history on dengue virus infection and pathogenicity
Source: Nat Commun. 2019 Mar 18;10:1246. doi: 10.1038/s41467-019-09193-y (PMC6423047; doi:10.1038/s41467-019-09193-y)
Supplement: Supplementary file 1 — Supplementary Information [file 41467_2019_9193_MOESM1_ESM.docx]

**Supplementary Information**

**for ‘ Effects of Infection History on Dengue Virus Infection and Pathogenicity’**

**Tsang *et al*.**

[1 SUPPLEMENTARY METHODS 2](#_Toc536720865)

[1.1 Construction of surveillance data from multiple sources 2](#_Toc536720866)

[1.2 Statistical model 3](#_Toc536720867)

[1.2.1 Overview 3](#_Toc536720868)

[1.2.2 Modeling the infection outcomes 4](#_Toc536720869)

[1.2.3 Modeling disease outcomes 7](#_Toc536720870)

[1.2.4 Left censoring of infection history 8](#_Toc536720871)

[1.2.5 Complete data joint likelihood 9](#_Toc536720872)

[1.3 Statistical inference: MCMC sampling scheme 10](#_Toc536720873)

[1.4 Simulation Study 12](#_Toc536720874)

[1.5 Exploring socioeconomic variables in data analysis 13](#_Toc536720875)

[1.6 Exploring effect of preseason antibody levels 14](#_Toc536720876)

[1.7 Goodness-of-fit 16](#_Toc536720877)

[2 Supplementary Notes 17](#_Toc536720878)

[2.1 Crude probability of diseases given infection 17](#_Toc536720879)

[2.2 Effects of prior infections and time since prior infections on the risk of a subsequent infection 18](#_Toc536720880)

[2.3 Software statement 18](#_Toc536720881)

[3 SUPPLEMENTARY REFERENCES 19](#_Toc536720882)

[4 SUPPLEMENTARY FIGURES 20](#_Toc536720883)

[5 SUPPLEMENTARY TABLES 25](#_Toc536720884)

# SUPPLEMENTARY METHODS

## Construction of surveillance data from multiple sources

We define an epidemic year as the time from July of one year to June of the next, with slight abuse of the word `epidemic' (some years are endemic). We obtained annual numbers of reported dengue cases in Nicaragua from the Pan American Health Organization (PAHO, http://www.paho.org) for calendar years 1995-2010. We also obtained from the Nicaragua Ministry of Health (NMOH) the numbers of dengue cases for the whole nation as well for Managua during epidemic years 2000-2010. Using these numbers, we calculated the proportions of the national case numbers accounted for by Managua in this period. We then multiplied the national case numbers from PAHO by these proportions to be the surveillance data for Managua during epidemic years 2000-2010. On average about 30% of all reported cases in Nicaragua occurred in Managua during epidemic years 2000-2010. Therefore, we multiply the PAHO case numbers during calendar years 1995-1999 by 0.3 to represent the surveillance case numbers in Managua for epidemic years 1995-1999.

Neither PAHO nor NMOH provide serotype-specific numbers of cases, but PAHO lists the co-circulating serotypes that were confirmed by virological surveillance in each nation. For epidemic years during the study period (04-05, 05-06, 06-07, 07-08, 08-09 and 09-10), serotype-specific case numbers are assumed proportional to the numbers of serotype-specific symptomatic infections detected by RT-PCR in the pediatric cohort in Managua. If a serotype was not detected by RT-PCR in the study year, we assume it accounted for 2% of the total number of surveillance-reported cases in that year. To construct serotype-specific surveillance case numbers in the years before the study, we first looked at which serotypes were circulating in each year as indicated by PAHO. To find out which serotype was dominant in Nicaragua in each epidemic year, we extracted information from published studies^1-4^. For each serotype that was neither mentioned by PAHO nor in the literature during a given year, we refer to it as neglectable and assume it accounted for 2% of total number of cases in that year. If a serotype was mentioned by PAHO but not designated by the literature as the dominant serotype in a given year, we refer to it as non-dominant and assume it accounted for 10% of total number of cases in that year. The remaining proportion was assigned to the dominant serotype specified in the literature. This allocation scheme is considered the primary analysis in our study, and hereafter referred to as Scenario 2. We conducted a sensitivity analysis by assuming either 5% (Scenario 1) or 15% (Scenario 3) for each non-dominant serotype. The surveillance data by serotype and year used in our analyses are summarized in Supplementary Figure 1.

## Statistical model

### Overview

We developed a Bayesian modeling framework that integrates the prospective Pediatric Dengue Cohort Study (PDCS) and the surveillance data in the study area. The cohort initiated its enrollment in August of 2004, and the data up to July of 2010 are included in this analysis. We index the study years by epidemic years 1-6, with year 1 corresponding to August of 2004 to June of 2005, year 2 corresponding to July of 2005 to June of 2006, and so on. Participants might enter or exit the study any time in the middle. At most seven serum samples were drawn from each participant in the months of July during 2004 to 2010 to determine possible inapparent infections. These serum-collection time points were also numbered by the years (1-6) with the first one numbered as 0. Let $T_{i0}$, $T_{i1}$ and $T_{i2}$ be the birth, entrance and exit year of person *i*. Let $\tilde{\mathbf{t}_{i}}=\{\tilde{t_{ij}}:j=1,\ldots,n_{i}\}$ be the vector of DENV infection years of person *i*, where $n_{i}\leq4$ is the number of infections. Let $\mathbf{V}_{i}=\{V_{ij}:j=1,\ldots,n_{i}\}$ be the corresponding serotypes of the infections. Let $R_{iv}(t)$ indicate whether person *i* is at risk to serotype *v* at the beginning of year *t.* Let $Y_{iv}(t)$ indicate infection (1) or no infection (0) of person *i* with regard to serotype *v* during year *t.* Let $S_{i}(t)$ indicate the disease (pathogenic) outcome of the host upon infection, either being inapparent (0) or having dengue-related clinical symptoms (1). More severe symptoms including dengue hemorrhagic fever and dengue shock syndrome are very rare and therefore not separately modeled. To increase identifiability of DENV infections, we make the following immunological assumptions:

1. A susceptible person can be infected by at most one serotype in each single year, i.e., $\sum_{1}^{4} Y_{iv}(t)\leq1$ for any given *i* and *t.*

2. Infection with a given serotype confers life-long immunity for that serotype, i.e., if person *i* is infected with serotype *v* in year *t,* this person is not at risk to that serotype for all year $\tau>t$.

There were two patients who had RT-PCR-confirmed co-infections of two serotypes, one patient in the 04-05 season and the other in the 05-06 season. To accommodate assumption (i), a single serotype was sampled from the co-infecting serotypes for each co-infection episode, and such sampling was repeated in the Bayesian framework. Although assumption (i) contradicts the biological possibility of co-infection in the same season, we expect such contradiction to have negligible impact on the validity of the modeling results as con-infection is indeed a rare event.

We have two stochastically dependent outcomes, the infection outcome, ${\{R}_{iv}\left( t \right),Y_{iv}\left( t \right)\}$ and the disease outcome $S_{iv}\left( t \right)$. Let $\mathbf{Y}_{i}\boldsymbol{(}t\boldsymbol{)}=\{Y_{iv}(t):v=1,2,3,4\}$. Let $\mathbf{H}_{i}^{Y}\left( t \right)$and $\mathbf{H}_{i}^{S}\left( t \right)$ be the history of infection and disease outcomes of person *i* up to year *t,* respectively. Infection history could refer to both $\mathbf{H}_{i}^{Y}\left( t \right)$ and $\mathbf{H}_{i}^{S}\left( t \right)$, but in our analysis, it refers to the former as the latter is not used as covariates to model the infection or disease outcomes. Let $\mathbf{H}_{i}^{X}\left( t \right)$ be the history of covariates that do not overlap with information provided in $\mathbf{H}_{i}^{Y}\left( t \right)$ and $\mathbf{H}_{i}^{S}\left( t \right)$, e.g., socioeconomic variables. A general presentation of the dengue model would be

(1)

$$P(\mathbf{H}_{i}^{Y}\left( T_{i2} \right),H_{i}^{S}\left( T_{i2} \right))=\prod_{{t=T}_{i0}}^{T_{i2}} \left\{ P\left( \mathbf{H}_{i}^{Y}\left( t \right) | \mathbf{H}_{i}^{Y}\left( t-1 \right) ,\mathbf{H}_{i}^{S}\left( t-1 \right),\mathbf{H}_{i}^{X}\left( t \right) \right)\times P\left( H_{i}^{S}\left( t \right) | \mathbf{H}_{i}^{Y}\left( t \right) ,\mathbf{H}_{i}^{S}\left( t-1 \right) ,\mathbf{H}_{i}^{X}\left( t \right) \right) \right\}=\prod_{{t=T}_{i0}}^{T_{i2}} \left\{ P\left( \boldsymbol{Y}_{\boldsymbol{i}}\boldsymbol{(t)} | \mathbf{H}_{i}^{Y}\left( t-1 \right) ,\mathbf{H}_{i}^{S}\left( t-1 \right) ,\mathbf{H}_{i}^{X}\left( t \right) \right)\times P\left( S_{i}(t) | \mathbf{H}_{i}^{Y}\left( t \right) ,\mathbf{H}_{i}^{S}\left( t-1 \right) ,\mathbf{H}_{i}^{X}\left( t \right) \right) \right\}$$

In practice, it is often more convenient to treat the history as part of the covariates. Define $\mathbf{X}_{i}^{Y}\boldsymbol{(t)}=\mathbf{X}^{Y}(\mathbf{H}_{i}^{Y}\left( t-1 \right) ,\mathbf{H}_{i}^{S}\left( t-1 \right) ,\mathbf{H}_{i}^{X}\left( t \right))$ as the covariates for infection outcomes synthesized from all relevant history. Similarly, define $\mathbf{X}_{i}^{S}\boldsymbol{(t)}=\mathbf{X}^{S}(\mathbf{H}_{i}^{Y}\left( t-1 \right) ,\mathbf{H}_{i}^{S}\left( t-1 \right) ,\mathbf{H}_{i}^{X}\left( t \right))$ as the covariates for disease outcomes. We discuss details in the probability structure for the two types of outcomes in the following sections.

### Modeling the infection outcomes

Define $T_{0}=\min\left( T_{i0} \right)$ and $T_{2}=\min\left( T_{i2} \right)$. In our analysis,$T_{0}=-8$ and $T_{2}=6$corresponding to epidemic years 95-96 and 09-10 respectively, and 1995 is the birth year of the oldest participant at enrollment of the study. Let $p_{v}\left( t \right)$ be the baseline probability that a DENV-naive person was infected with serotype *v* in year *t,* *v*=1,2,3,4*,* $T_{0}\leq t\leq T_{1}$*.* The effective probability of infection adjusted for covariates $\mathbf{X}_{i}^{Y}\left( t \right)$ is:

(2)

$$log\frac{p_{iv}(t)}{1-p_{iv}(t)}=log\frac{p_{v}(t)}{1-p_{v}(t)}+\mathbf{X}_{i}^{Y}\left( t \right)'\boldsymbol{\beta}^{Y}$$

where $\boldsymbol{\beta}^{Y}$ is the vector of covariate coefficients, and $\mathbf{X}_{i}^{Y}\left( t \right)'$ is the transpose of the covariate vector $\mathbf{X}_{i}^{Y}\left( t \right)$.

We assume competing risks among the four serotypes within each year. Assuming the hazards of all serotypes are proportional to each other within each year, it can be shown that, given infection with any serotype in year *t,* the probability that individual *i* is infected by serotype *v* is:

(3)

$$\gamma_{iv}\left( t \right)=\frac{{\log\left[ 1-p_{iv}\left( t \right) \right]}^{R_{iv}(t)}}{\sum_{u=1}^{4} {\log\left[ 1-p_{iv}\left( t \right) \right]}^{R_{iv}(t)}}$$

The probability of person *i* escaping infection in year *t* from all serotypes to which the person is at risk is:

(4)

$$q_{i}\left( t \right)=\prod_{v=1}^{4} \left[ 1-p_{iv}\left( t \right) \right]^{R_{iv}(t)}$$

Let $\mathbf{p}=\{p_{v}(t):v=1,2,3,4,T_{0}\leq t\leq T_{2}\}$. Let $Y_{i*}(t)=max(R_{iv}\left( t \right)*Y_{iv}\left( t \right),v=1,2,3,4)$, which indicates infection of person *i* with any serotype during year *t,* The probability of infection outcomes for person-year*(i,t)* is:

(5)

$$P\left( \mathbf{Y}_{i}\left( t \right) | \mathbf{X}_{i}^{Y}\left( t \right)\boldsymbol{,}\mathbf{p}\boldsymbol{,}\boldsymbol{\beta}^{Y} \right)=q_{it}^{1-Y_{i*}(t)}\left\{ \left[ 1-q_{i}\left( t \right) \right]\prod_{v=1}^{4} \gamma_{iv}\left( t \right)^{Y_{iv}\left( t \right)} \right\}^{Y_{i*}(t)}$$

The following covariates are used to adjust the baseline infection probability:

1. $X_{i1}^{Y}(t)$: indicator for age group, ≤8 years (0) and >8 years (1).

2. $X_{i2}^{Y}\left( t \right),X_{i3}^{Y}(t)$: indicators for the number of prior infections

- $X_{i2}^{Y}\left( t \right)=X_{i3}^{Y}\left( t \right)=0$: DENV-naive

- $X_{i2}^{Y}\left( t \right)=1,X_{i3}^{Y}\left( t \right)=0$: one prior infection

- $X_{i2}^{Y}\left( t \right)=0,X_{i3}^{Y}\left( t \right)=1$: two or more prior infections

3. $X_{i4}^{Y}\left( t \right),X_{i5}^{Y}(t)$: indicators for time since last infection among individuals having one prior infection.

- $X_{i4}^{Y}\left( t \right)=X_{i5}^{Y}\left( t \right)=0$: one year

- $X_{i4}^{Y}\left( t \right)=1,X_{i5}^{Y}\left( t \right)=0$: two years

- $X_{i4}^{Y}\left( t \right)=0,X_{i5}^{Y}\left( t \right)=1$: three or more years

Note that “one year” here refers to the epidemic year after the infection year and does not mean exactly one year (12 months) after the day of infection. This is because infections were identified by annual serological samples, and we cannot pinpoint the exact infection time. The actual time between infection and any time the epidemic year after the infection year could vary from 1 to 24 months. Even if we consider that the epidemic season in Nicaragua is mostly August-January, the actual time span could still vary from 6-18 months. Similarly, “two years” refers to the second epidemic year after the infection year, and so on.

4. $X_{i6}^{Y}\left( t \right),X_{i7}^{Y}(t)$: indicators for time since last infection among individuals having two or more prior infection.

- $X_{i6}^{Y}\left( t \right)=X_{i7}^{Y}\left( t \right)=0$: one year

- $X_{i6}^{Y}\left( t \right)=1,X_{i7}^{Y}\left( t \right)=0$: two years

- $X_{i6}^{Y}\left( t \right)=0,X_{i7}^{Y}\left( t \right)=1$: three or more years

5. $X_{i8}^{Y}(t)$: indicator for home ownership.

6. $X_{i9}^{Y}\left( t \right),X_{i10}^{Y}(t)$,$X_{i11}^{Y}\left( t \right),X_{i12}^{Y}(t)$: indicators for ownership of electric fans in the house

- All equal to 0: having no fan

- Only $X_{i9}^{Y}\left( t \right)=1$: 1-2 fans

- Only $X_{i10}^{Y}\left( t \right)=1$: 3-4 fans

- Only $X_{i11}^{Y}\left( t \right)=1$: 5-6 fans

- Only $X_{i12}^{Y}\left( t \right)=1$: ≥7 fans

### Modeling disease outcomes

Logistic form is used to adjust the baseline probability of developing symptoms given infection. Let $\phi_{v}$ be the baseline probability of disease given infection with serotype *v.* The effective probability of disease in infection year  $\tilde{t_{ik}}$ of individual *i* is:

(6)

$$log\frac{\phi_{iv}(t)}{1-\phi_{iv}(t)}= \sum_{v=1}^{4} I\left( V_{ik}=v \right) [log\frac{\phi_{v}(t)}{1-\phi_{v}(t)}+\mathbf{X}_{i}^{S}\left( \tilde{t_{ik}} \right)'\boldsymbol{\beta}^{S}]$$

where $\beta^{S}$ is the vector of covariate effects on pathogenicity. Let $\boldsymbol{\phi}=\{\phi_{v}:v=1,2,3,4\}$.

The probability of disease outcomes for person-year $(i, \tilde{t_{ik})}$ is:

(7)

$$P\left( S_{i}\left( t \right)|\mathbf{X}_{i}^{S}\left( t \right),\boldsymbol{\phi},\boldsymbol{\beta}^{S} \right)=\left\{ \begin{aligned} \phi_{ik}^{S_{i}\left( t \right)}\left( 1-\phi_{ik} \right)^{1-S_{i}(t)}, t=\tilde{t_{ik}} ,k=1,2,3,4, \\ 0, \mathrm{otherwise}. \end{aligned} \right.$$

The following covariates are used to adjust the baseline probability of developing clinical disease given infection:

1. $X_{i1}^{S}(t)$: indicator for age group, ≤8 years (0) and >8 years (1). We chose the cut-off of 8 years old because dengue vaccine is licensed for individuals aged 9 years or older (<http://www.who.int/immunization/research/development/dengue_q_and_a/en/>) and we hope to provide relevant information for future research on vaccine design or vaccination strategies.

2. $X_{i2}^{S}\left( t \right),X_{i3}^{S}(t)$: indicators for time since last infection among children ≤8 years old

- $X_{i2}^{S}\left( t \right)=X_{i3}^{S}\left( t \right)=0$: one year

- $X_{i2}^{S}\left( t \right)=1,X_{i3}^{S}\left( t \right)=0$: two years

- $X_{i2}^{S}\left( t \right)=0,X_{i3}^{S}\left( t \right)=1$: three or more years

3. $X_{i4}^{S}\left( t \right),X_{i5}^{S}(t)$: indicators for time since last infection among children >8 years old

- $X_{i4}^{S}\left( t \right)=X_{i5}^{S}\left( t \right)=0$: one year

- $X_{i4}^{S}\left( t \right)=1,X_{i5}^{S}\left( t \right)=0$: two years

- $X_{i4}^{S}\left( t \right)=0,X_{i5}^{S}\left( t \right)=1$: three or more years

4. $X_{i6}^{S}\left( t \right),X_{i7}^{S}(t)$: indicators for number of prior infection among children ≤8 years old

- $X_{i6}^{S}\left( t \right)=X_{i7}^{S}\left( t \right)=0$: no prior infection

- $X_{i6}^{S}\left( t \right)=1,X_{i7}^{S}\left( t \right)=0$: one previous infection

- $X_{i6}^{S}\left( t \right)=0,X_{i7}^{S}\left( t \right)=1$: two of more previous infections

5. $X_{i8}^{S}\left( t \right),X_{i9}^{S}(t)$: indicators for number of prior infection among children >8 years old

- $X_{i8}^{S}\left( t \right)=X_{i9}^{S}\left( t \right)=0$: no prior infection

- $X_{i8}^{S}\left( t \right)=1,X_{i9}^{S}\left( t \right)=0$: one previous infection

- $X_{i8}^{S}\left( t \right)=0,X_{i9}^{S}\left( t \right)=1$: two of more previous infections

### Left censoring of infection history

The infection history of each participant was left-censored at study enrollment, but individual level data can provide some information. The following guidelines based on individual data are used to inform the enumeration of possible infection history before study enrollment:

1. If an infection of a serotype occurred during the study period, prior infection of that serotype is considered impossible.

2. If baseline neutralization titration (NT) assay using reporter virus particles is negative for a serotype, prior infection by that serotype before baseline is considered to be impossible. Otherwise, the individual is considered to be infected by that serotype before baseline. If NT is not available, go to next.

3. If PRNT≤20 for a serotype, prior infection by that serotype before baseline is considered impossible. Otherwise, the individual is considered to be infected by that serotype before baseline. If PRNT is not available, go to next.

4. If baseline inhibition ELISA (iELISA) is seronegative, the individual is considered dengue-naive at baseline. Otherwise, the individual is considered to have at least one prior infection.

In the probability setting, inference on the left-censored infection history can be further helped with historic dengue surveillance data at the population level. The basic idea is that, the relationship between the surveillance-reported case numbers and the infection numbers observed in the cohort during the study period can provide information about the unobserved infection numbers in the cohort conditioning on surveillance-reported case numbers during the pre-study years.

Let $M_{v}(t)$ be the observed number of surveillance-reported clinical cases in Managua and $m_{v}(t)$ be the number of infections in the study cohort for serotype $v:v=1,2,3,4$ in year $t:T_{0}\leq t\leq T_{2}$. Let $n_{t}$ be the study population size in year *t* and *N* be the total population size in Managua. Note that both $m_{v}(t)$ and $n_{t}$ are well defined for the study cohort during pre-study years by tracing the time backward. Let $\rho$ be the proportion of DENV infections in the area that can be captured by surveillance. Let *c* be the risk ratio between children of 2-14 years old and the general population in Managua. We assume that, conditioning on $m_{v}(t)$, $M_{v}\left( t \right)\sim Poisson(\frac{Nm_{v}\left( t \right)}{c n_{t}}\rho)$ , where $\frac{Nm_{v}\left( t \right)}{c n_{t}}$ is the expected number of DENV infections at the municipality level obtained by scaling the number of infections in the study cohort. Municipal population size *N,* risk ratio *c* and surveillance capture proportion $\rho$ are assumed to be time-independent. Here we ignore the changes in the age structure of the study cohort when we trace back in time, which is reasonable because the variation of the risk of infection within the age group of 2-14 years old is relatively small as compared to that in the general population^5^. As the data contain no information to identify $\rho$ and *c* separately, $\rho/c$ is treated as a single unknown parameter and will be replaced by $\rho$ hereinafter.

It makes more intuitive sense to link $M_{v}\left( t \right)$ to the number of symptomatic infections in the study cohort. However, simulation studies suggest that such a link will lead to nonidentifiability of pathogenicity-related parameters. As a result, we link $M_{v}\left( t \right)$to $m_{v}\left( t \right)$the total number of infections in the study cohort.

### Complete data joint likelihood

Let $\mathbf{m}=\left\{ m_{v}\left( t \right),1\leq v\leq4,T_{0}\leq t\leq T_{2} \right\}$ and $\mathbf{M}=\{M_{v}\left( t \right),1\leq v\leq4,T_{0}\leq t\leq T_{2}\}$. Let $\pi(\boldsymbol{\psi})$ be the joint prior for all the parameters $\boldsymbol{\psi}=(\mathbf{p},\boldsymbol{\phi},\boldsymbol{\beta}^{Y},\boldsymbol{\beta}^{S},\rho)$ .The complete-data joint probability is:

$$P\left( \boldsymbol{\psi,Y,S,}\mathbf{X}^{Y}\mathbf{,}\mathbf{X}^{S}\mathbf{,M} \right)=\prod_{t=T_{0}}^{T_{2}} \prod_{i=1}^{n_{t}} \left\{ q_{i}\left( t \right)^{1-Y_{i*}\left( t \right)}\left[ \left( 1-q_{i}\left( t \right) \right)\prod_{v=1}^{4} \left( \gamma_{iv}\left( t \right)\phi_{iv}^{S_{i}\left( t \right)}\left( 1-\phi_{iv} \right)^{1-S_{i}\left( t \right)} \right)^{Y_{iv}\left( t \right)} \right]^{Y_{i*}(t)} \right\}\times\prod_{t=T_{0}}^{T_{2}} \prod_{v=1}^{4} f_{\mathrm{pois}}\left( M_{v}\left( t \right) | \frac{m_{v}\left( t \right)}{n_{t}/N}\rho\right)\times\pi(\boldsymbol{\psi})$$

where $f_{\mathrm{pois}}(.|\mu)$ is the probability mass function of Poisson distribution with mean $\mu$.

(8)

A schematic for the hierarchical modeling framework is shown in Supplementary Figure 4 to help readers understand how different components of the model are related to each other. This schematic is based on the data generation or dependence mechanism, following the general logic flow (indicated by the arrows) that components of the model take parameter as inputs and generate the individual-level infection histories which then turn into observed data by study follow-up and lab assays. The infection histories themselves together with demographic and socioeconomic variables form covariates for subsequent risks of infection and pathogenicity. The infection histories are also need as direct inputs for the models for pathogenicity and surveillance, because (1) dengue disease occurs only if infection occurs, and (2) per our assumption surveillance counts depend on serotype-specific annual numbers of infections that are aggregated from infection histories. The outcomes of the model are posterior samples of parameters and the infection histories, denoted by dashed boxes.

## Statistical inference: MCMC sampling scheme

We use flat priors for all parameters. For probability parameters, we use uniform(0,1) as the prior. For regression coefficients, we use uniform(-10,10) as the prior.

There are two types of missing data in our study. First, infections and associated disease outcomes that occurred before study enrollment are unobserved. Second, for the infections that are confirmed by iELISA during the study period, the responsible serotypes are unknown. These unobserved infection and disease outcomes are sampled in the MCMC procedure. We refer to a possible sequence of infection and disease history from birth year to the end of the study year for an individual as a pathway. We first enumerate all possible pathways that are compatible with observed infection outcomes during the study years and the baseline serology data under the immunological assumptions guidelines listed in Sections 2.1 and 2.4 (hereinafter referred to as compatible pathways). At the initial point, we randomly assigned one possible infection path to each participant.

In each MCMC step, we first sample each model parameter via a random walk Metropolis-Hastings algorithm. Take $p_{v}\left( t \right)$ for example. A new value $p_{v}^{*}\left( t \right)$ is proposed from $f_{\mathrm{Normal}}(p_{v}\left( t \right),\sigma_{vt}^{2})$, which is the normal density with mean $p_{v}\left( t \right)$ and variance $\sigma_{vt}^{2}.$The new value is accepted with probability:

(9)

$$\alpha=min\left\{ 1,\frac{P(\boldsymbol{\psi}^{*},\mathbf{Y,S,}\mathbf{X}^{Y}\mathbf{,}\mathbf{X}^{S}\mathbf{,M})}{P(\boldsymbol{\psi},\mathbf{Y,S,}\mathbf{X}^{Y}\mathbf{,}\mathbf{X}^{S}\mathbf{,M}} \right\}$$

where $\boldsymbol{\psi}^{*}$ is the parameter vector with $p_{v}\left( t \right)$ replaced by $p_{v}^{*}\left( t \right)$.

The sampling of infection history (infection outcomes) for one individual is not independent of that for others, as the total numbers of serotype-specific infections in the cohort during each year are influenced by the surveillance data. For individual *i,* let $K_{i}$ be the number of compatible pathways, which are represented by $\left( \mathbf{Y}_{i}^{\left( k \right)},\mathbf{S}_{i}^{\left( k \right)},\mathbf{X}_{i}^{Y\left( k \right)},\mathbf{X}_{i}^{S\left( k \right)} \right),k=1,2,\ldots,K_{i}$. As the total number of infections *m* in the cohort may change according to the individual-level pathway, let $m^{(k)}$ be the value of *m* corresponding to $\left( \mathbf{Y}_{i}^{\left( k \right)},\mathbf{S}_{i}^{\left( k \right)},\mathbf{X}_{i}^{Y\left( k \right)},\mathbf{X}_{i}^{S\left( k \right)} \right),k=1,2,\ldots,N_{i}$. At MCMC step *j,* for each compatible pathway of individual *i,* we calculate the likelihoods:

(10)

$$P\left( \boldsymbol{\psi}^{\left( j \right)}\boldsymbol{,}\mathbf{Y}_{i}^{\left( k \right)}\boldsymbol{,}\mathbf{S}_{i}^{k}\boldsymbol{,}\mathbf{X}_{i}^{Y(k\boldsymbol{)}}\boldsymbol{,}\mathbf{X}_{i}^{S\left( k \right)}\boldsymbol{,}\mathbf{M}^{\boldsymbol{(}k\boldsymbol{)}} \right)=\prod_{t=T_{0}}^{T_{2}} \{P\left( Y_{i}^{\left( k \right)}\left( t \right) | X_{i}^{Y\left( k \right)}\left( t \right),\mathbf{p}^{\left( j \right)},\boldsymbol{\beta}^{Y\left( j \right)} \right)\times P(S_{i}^{\left( k \right)}(t)|X_{i}^{S\left( k \right)}\left( t \right),\boldsymbol{\phi}^{\left( j \right)},\boldsymbol{\beta}^{S(j)})\}\times\prod_{t=T_{0}}^{T_{2}} \prod_{v=1}^{4} f_{\mathrm{pois}}\left( M_{v}\left( t \right) | \frac{m_{v}^{(k)}\left( t \right)}{n_{t}/N}\rho^{(j)} \right)$$

where $k=1,2,\ldots.,K_{i}$, $\boldsymbol{\psi}^{(j)}=(\mathbf{p}^{\left( j \right)},\boldsymbol{\phi}^{\left( j \right)},\boldsymbol{\beta}^{Y\left( j \right)},\boldsymbol{\beta}^{S\left( j \right)},\rho^{(j)})$ is the current parameter vector. We then sample a compatible pathway for individual *i* using these likelihoods as the weights. In this sampling approach, all compatible pathways are enumerated based on potential infection and disease outcomes of all four serotypes, which could be computationally impractical if the number pre-study years is large. We refer to this sampling approach as the 4-serotype sampling. Alternatively, we can sample pathways formed by two randomly selected serotypes each time, conditional on the current setting of the other two serotypes. We refer to this sampling scheme as the 2-serotype sampling. The least computational burden is offered by sampling a single serotype at a time, i.e., forming compatible pathways based on a single serotype, conditioning on the current setting of the other three serotypes. At each MCMC step, the four serotypes are arranged in a random order, and the updating proceeds one by one. We refer to this sampling schemes as the 1-serotype sampling. However, it can be shown that the 1-serotype sampling results in biased estimates, because when the infection outcomes of the other three serotypes are fixed, the infection outcome of the remaining serotype could have very limited choices due to immunological constraints, which will make the jumping across the actually compatible pathways of all four serotypes very inefficient or even stuck locally. We compare the performance of the three sampling approaches in simulation.

## Simulation Study

We simulated infection and disease processes according to the model structure in a hypothetical cohort of size 2000. Annual infection probabilities and the probabilities of developing disease given infection (pathogenicity) were set to be serotype-specific, whereas covariate effects for infection risk and pathogenicity were shared across serotypes. All individuals were set to enter the study at the same time with ages at enrolment randomly assigned to 2-5 years, and to be followed for six years. The probability of missing serotype information was set to 0.6 for each infection during the study years. The disease outcomes (symptomatic vs. inapparent) were set to be known during the study and unknown before the study.

The true values for the annual probabilities of infection for each year and serotype were set to range from 0.05 to 0.20. We set $\boldsymbol{\phi}=(0.3,0.2,0.4,0.1)$, $\boldsymbol{\beta}^{Y}=(1,1.5,0.5,0.4,-0.5)$, and $\boldsymbol{\beta}^{S}=(-0.5,-1,-0.1,0.2)$**.** The covariates considered are the number of prior infections (0, 1 and ≥2 indicators), years since the most recent infection (1, 2 and ≥3 indicators) for both the risk of infection and the risk of disease given infection, as well as age group for the risk of infection ≤8 and >8, 1 indicator). A total of 100 simulations were conducted.

The comparison between the three different sampling approaches is summarize in Supplementary Figure 3. The 1-serotype sampling clearly resulted in much more biased estimates for many of the annual infection probabilities and probabilities of disease given infection during the study period, as compared to the other two. Interestingly, not much difference in the estimated covariate effects for either infection or pathogenicity was found between the sampling approaches. The performances of the 2-serotype sampling and 4-serotype sampling are very similar, both providing nearly unbiased estimates for most model parameters and intervals formed by 2.5% and 97.5% of those estimates from the 100 simulated epidemics covering all parameters.

## Exploring socioeconomic variables in data analysis

From initial analysis that did not include any covariate other than infection history for the infection probabilities, we found that the probability of infection increases with two or more prior infections. We then suspected that there was a selection bias such that children with repeated infections might have been much more extensively exposed than others. In an effort to reduce such bias, we adjusted the infection probabilities for individual-level socioeconomic variables, including parent education level, floor type of the house, and ownership of house, car, television sets, electric fans, refrigerators, and animals. These variables were collected in household surveys administered during 2008-2010. The results of screening analysis, which looks at Spearman correlation between infection with any DENV and each variable, were summarized in Supplementary Table 5. We found that home (house) ownership and possession of electric fans were negatively associated with infection, with p-value < 0.05. The p-values for school type and mother’s education were also close to 0.05. Mother education was not considered because only the “university” category differs from other categories in incidence of DENV infection, but this category has a small number of children. We fitted a logistic regression of observed DENV-infection status during the study period on school type, home ownership and number of fans, where private and semi-private were combined for school type, and number of fans was treated as continuous. School type was not significant (p-value=0.5) in the presence of home ownership (p-value=0.014) and number of fans (p-value=0.032). As a result, we think the number of fans and household ownership best represent the social economic variables that may be predictive of DENV infection.

## Exploring effect of preseason antibody levels

Using the MCMC samples that provide information on the infection and disease outcome for each individual from birth, we were able to explore the association between pre-season antibody levels and probability of infection and probability of disease given infection after primary infection. For each MCMC iteration selected for joint inference, we extracted secondary infections with preseason antibody levels reflected by either PRNT, NT or iELISA during the study period. We then fitted a logistic regression of the disease outcomes of secondary infections (all non-primary infections) on three types of preseason antibody levels: homologous and heterologous neutralizing antibodies measured by PRNT or NT and the total dengue-specific binding antibodies measured by iELISA. We did not use the levels of antibody as continuous variables because the relationship between antibody and probability of infection or disease given infection could be non-linear and non-monotonic, according to previous studies^6^.

We estimate those effects as follows: Suppose $\hat{\beta}^{(j)}$ is the estimate of parameter $\beta$ in the logistic regression using the *j^th^* MCMC sample, and let ${s^{2}(\hat{\beta}}^{(j)})$ be the variance of $\hat{\beta}^{(j)}$ estimated by the logistic regression. With *J*=5000 effective MCMC samples, the final estimate of $\beta$ is $\bar{\hat{\beta}}=\frac{1}{j}\sum_{j=1}^{J} \hat{\beta}^{(j)}$, and the variance of the final estimate is:

(11)

$$\mathrm{Var}\left( \bar{\hat{\beta}} \right)=\frac{1}{J}\sum_{j=1}^{J} \left( \hat{\beta}^{\left( j \right)}-\bar{\hat{\beta}} \right)^{2}+\frac{1}{J}\sum_{j=1}^{J} {s^{2}(\hat{\beta}}^{(j)})$$

which accounts for uncertainty from both the regression and the MCMC sampling. The results were summarized in Supplementary Table 7 and were described in the main text. In each logistic regression, age, number of prior infections, epidemic season were adjusted for.

For iELISA, we observed that low titers (10-80) were associated with about 2-fold increase in the risk of symptomatic infection, compared to preseason seronegative (<10) individuals (Supplementary Table 7), consistent with the findings in Katzelnick et al.^7^, although our results did not reach statistical significance. Our model permits the decomposition of the effect of iELISA into the effects on the risk of infection and the risk of disease given infection. Interestingly, the risk of infection was inversely correlated with the iELISA titer among preseason seropositive children. Relative to seronegatives, iELISA titers between 10 and 80 were associated with a 2- to 3-fold increase in the risk of secondary infection, whereas titers above 1280 were associated with more than 60% decrease in the risk of secondary infection. In terms of the probability of disease given secondary infection, the risk associated with of low to medium level of titers (10-320) remained similar to the seronegatives. On the other hand, high titers (>1280) were associated with more than 3-fold increase in the risk. This surprising pathogenicity-enhancing effect of high iELISA titers could be due to the possibility that, when the preseason titer was already high, inapparent infections might not be able to induce a 4-fold increase of iELISA titer in paired sera and might thus have been under-detected, which in turn artificially raised the proportion of disease. As a sensitivity analysis, we relaxed the definition of infection such that a 2-fold increase in iELISA also implies infection when the preseason titer was ≥1280. The results in Supplementary Table 8 indicate that the pathogenicity-enhancing effect of high titers indeed disappeared. As expected, the protective effect of high titers against infection also vanished.

For neutralizing antibodies, we did not explore the association between antibody levels and probability of secondary infection, mainly because the selection of samples to undergo PRNT or NT was not random and mostly from children with repeated infections as detected by iELISA, which may potentially bias the estimation. Therefore, we only assessed the association between neutralizing antibody levels and probability of disease given secondary infection. The low, medium and high levels for serotype-specific neutralizing antibodies, measured jointly by percent inhibition of single-dilution PRNT and titers of NT, are defined as follows. We first categorize PRNT and NT:

PRNT: low (≤8), medium (9-39) and high (>39), and

NT: low (≤5), medium (6-74) and high (>74),

where the cut points are determined by their 33% and 66% quantiles. The higher category between PRNT and NT is then used to indicate the antibody level. For example, if PRNT is high and NT is low for a given sample, the antibody level is high for that sample. The level of the preseason antibody specific to the same (different) serotype as the infecting one is referred to as homologous (heterologous). For homologous antibody, the odds ratio was estimated to be 1.17 (95% CI: 0.33, 4.20) for the medium and 1.11 (95% CI: 0.32, 3.83) for the low level respectively, compared to the high level. For heterologous antibody, the odds ratio was estimated to be 0.99 (95% CI: 0.22, 4.51) for the medium and 0.81 (95% CI: 0.07, 9.20) for the low level respectively, compared to the high level. In conclusion, we found no statistical association between the level of neutralizing antibody and the probability of disease given secondary infection.

## Goodness-of-fit

We evaluated the goodness-of-fit of our fitted model via a simulation study. We simulated dengue epidemics in the pediatric cohort using a subset of the posterior samples of the parameters. We then compared the non-serotype-specific annual attack rates (proportions of infected individuals in each year) during 2004-2009 to the observed values. A total of 10000 dengue epidemics were simulated, each based on a separate draw of parameters from their posterior joint distribution. The median (50%) as well as the 2.5% and 97.5% quantiles of the simulated annual attack rates were reported in Supplementary Table 9. The median attack rates are similar to the observed ones and the (2.5%, 97.5%) quantile intervals cover the observed proportion, suggesting that our model fits the observed data reasonably well.

For infectious disease data, goodness-of-fit can be formally tested in a conditional fashion, that is, conditioning on the past, observed numbers of infections are compared with model-predicted frequencies^8^. For each posterior sample of infection histories and parameters, we calculate the model-predicted probability of infection (regardless of serotype) for each individual during each study year *t*, conditioning on the sampled infection history of that individual up to year *t* -1. These probabilities are then summed over the study population to obtain model-predicted total number of dengue infections during year *t* conditioning on the current posterior sample. Averaging these model-predicted annual total numbers of infections over all posterior samples, we obtain the model-predicted numbers conditioning on observed infection history, i.e., uncertainty in the infection histories has been integrated out. We then form the test statistic $\sum_{t=1}^{6} \frac{{(n_{t}-\hat{n}_{t})}^{2}}{\hat{n}_{t}}$ , where $n_{t}$ is the observed annual numbers of infections during year *t* and $\hat{n}_{t}$ is the model-predicted counterpart conditioning on observed history. As some children were enrolled in the middle of the study, infection status of these children in the early study years was not observed. We used the posterior average of their unobserved infection status during the study in the calculation of $n_{t}$. Following the goodness-of-fit test for logistic models^9^, the test statistic follows a chi-squared distribution, $\chi_{4}^{2}$, under the null hypothesis that there is no lack of goodness-of-fit. The p-value is 0.997, suggesting that the model fits the data very well. Further stratification of the study population by age group (2-8 years old and >8 years old) in each is associated with a p-value of 0.36 (based on $\chi_{10}^{2}$), also suggesting a decent fit. The observed and model-predicted annual numbers of infections, stratified by age group or not, are shown in Supplementary Table 10.

# Supplementary Notes

## Crude probability of diseases given infection

As most information about pathogenicity comes from the cohort data during the study period, we expect consistency between the model estimates about pathogenicity and those indicated by the raw data. We computed crude probabilities of diseases given infection (Supplementary Table 12) stratified by age, sex and infection history. For infection history, we assume that baseline seropositive children had been infected exactly once before baseline (study entry). Our model estimates are consistent with strong signals in these crude pathogenicity results. For example, pathogenicity in children older than 8 years was much higher than younger children regardless of baseline serostatus, 29% vs. 18% among baseline seronegatives and 24% vs. 12% among baseline seropositives. Another example is the lower risk of disease given infection among children >8 years old at 2 years after the most recent infection compared to 1 year after, 6% vs. 28% among baseline seronegatives and 14% vs. 33% among baseline seropositives. Supplementary Table 2 provides some information about relative pathogenicity among the serotypes. Suppose that PCR captured all symptomatic infections, and that we can allocate ELISA-captured inapparent (asymptomatic) infections to the serotypes according to the proportions of serotype-specific inapparent infections captured by NT and PRNT. Then, compared to DENV-1, DENV-3 had a crude odds ratio of 4.25 (95% confidence interval [CI]: 2.98, 6.12) for disease given infection, and the crude odds ratios were 1.53 (95% CI: 1.07, 2.21) for DENV-2 and 0.038 (95% CI: 0001, 0.23) for DENV-4. A similar pattern is seen in the corresponding model-estimated odds ratios, 2.84, 1.34 and 0.18. The differences in exact magnitudes are likely a result of the adjustment for age and infection history in the model.

## Effects of prior infections and time since prior infections on the risk of a subsequent infection

Our model contains interactions between covariates, and interpretation of the effect of one covariate is thus conditional on other covariates. For example, how the time since the most recent infection affected the risk of infection depends on whether the most recent infection was a primary infection or not (Figure 2 in the main text). However, reorganization of some of the model estimates could provide more intuitive interpretation. For example, compared to no prior infection, the odds ratios for the risk of infection at 1, 2 and >2 years after the primary infection are 0.46, 0.46×1.12=0.52, and 0.46×1.47=0.68, respectively (numbers from scenario 2 in Supplementary Table 4), suggesting that in general one prior infection was protective against a subsequent infection and the protection seems to decay over time.

Similarly, for >1 prior infections, the odds ratios at 1, 2 and >2 years after the most recent infection are 1.91, 1.91×0.76=1.45, 1.91×0.66=1.26 in reference to no prior infection, suggesting that two or more prior infections boosted the risk a subsequent DENV infection, but such boosting effect weakened over time.

## Software statement

All simulation and data analyses were performed using R 3.2.5, and C++ was implemented using the R package Rcpp.^10^

# SUPPLEMENTARY REFERENCES

1. Hammond, S. N. *et al.* Differences in dengue severity in infants, children, and adults in a 3-year hospital-based study in Nicaragua. *Am J Trop Med Hyg* **73**, 1063-1070 (2005).

2. Harris, E. *et al.* Typing of dengue viruses in clinical specimens and mosquitoes by single-tube multiplex reverse transcriptase PCR. *J Clin Microbiol* **36**, 2634-2639 (1998).

3. Harris, E. *et al.* Clinical, epidemiologic, and virologic features of dengue in the 1998 epidemic in Nicaragua. *Am J Trop Med Hyg* **63**, 5-11 (2000).

4. OhAinle, M. *et al.* Dynamics of dengue disease severity determined by the interplay between viral genetics and serotype-specific immunity. *Sci Transl Med* **3**, 114ra128 (2011).

5. Egger, J. R. & Coleman, P. G. Age and clinical dengue illness. *Emerg Infect Dis* **13**, 924-925 (2007).

6. Katzelnick, L. C. *et al.* Antibody-dependent enhancement of severe dengue disease in humans. *Science* **358**, 929-932 (2017).

7. Katzelnick, L. C., Montoya, M., Gresh, L., Balmaseda, A. & Harris, E. Neutralizing antibody titers against dengue virus correlate with protection from symptomatic infection in a longitudinal cohort. *Proc Natl Acad Sci U S A* **113**, 728-733 (2016).

8. Yang Y, Longini, IM and Halloran, ME. Design and Evaluation of Prophylactic Intervention Using Infectious Disease Incidence Data from Close Contact Groups. *Journal Of the Royal Statistical Society, Series C*. **55**, 317-330 (2006).

9. Hosmer,D.W. and Lemeshow, S. Goodness of fit tests for themultiple logistic regression model. *Communs Statist. Theory Meth*. **9**, 1043–1069 (1980).

10. R Core Team. R: A language and environment for statistical computing. Vienna, Austria: R Foundation for Statistical Computing, 2016. Available at: http://www.R-project.org/

# SUPPLEMENTARY FIGURES

**
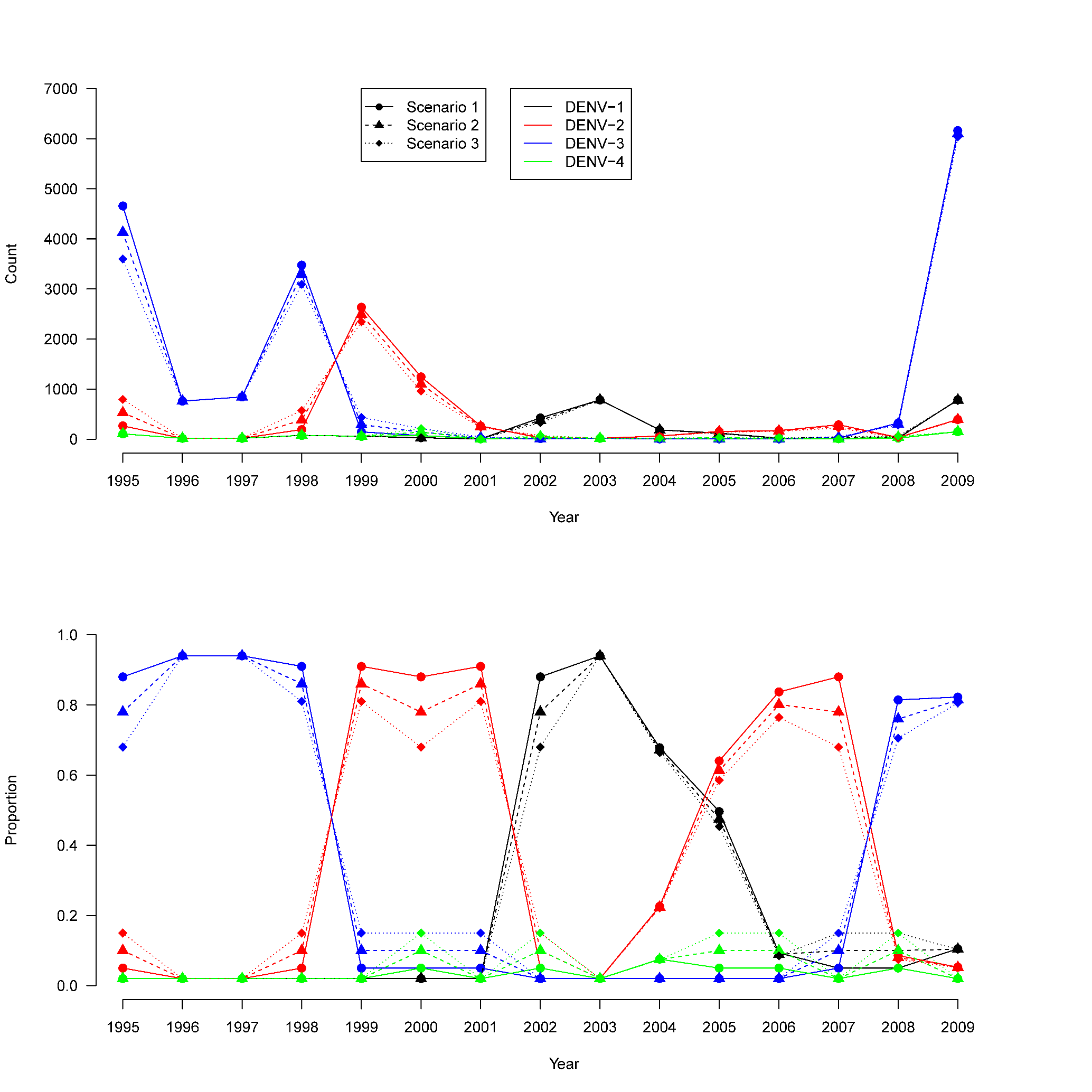
**

**Supplementary Figure 1.** Summary of the surveillance data used in the analyses. Scenarios 1, 2 and 3 correspond to the assumptions of 5%, 10% and 15%, respectively, as the proportion of non-dominant serotypes (reported by virological surveillance) among all surveillance-reported cases for the pre-study years (Supplementary Methods, Section 1.1). Source data are provided as a Source Data file.


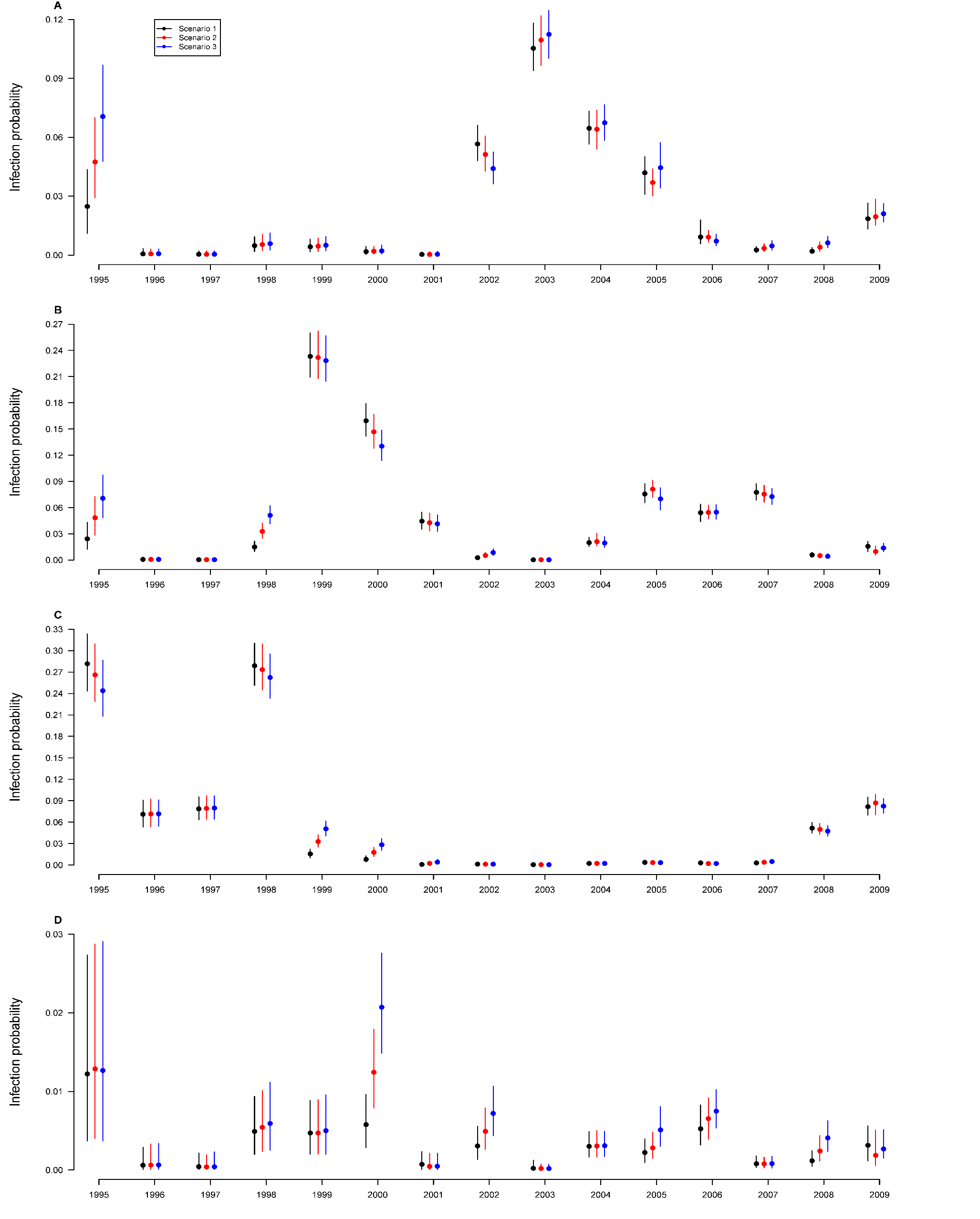


**Supplementary Figure 2.** Posterior medians (points) and 95% credible intervals (vertical bars) for the annual probabilities of infection from epidemic year 1995 (95-96 season) to epidemic year 2009 (09-10 season) for DENV-1 (panel A), DENV-2 (panel B), DENV-3 (panel C) and DENV-4 (panel D). Scenarios 1, 2 and 3 correspond to the assumptions of 5%, 10% and 15%, respectively, as the proportion of non-dominant serotypes (reported by virological surveillance) among all surveillance-reported cases for the pre-study years (Supplementary Methods, Section 1.1). Source data are provided as a Source Data file.

**
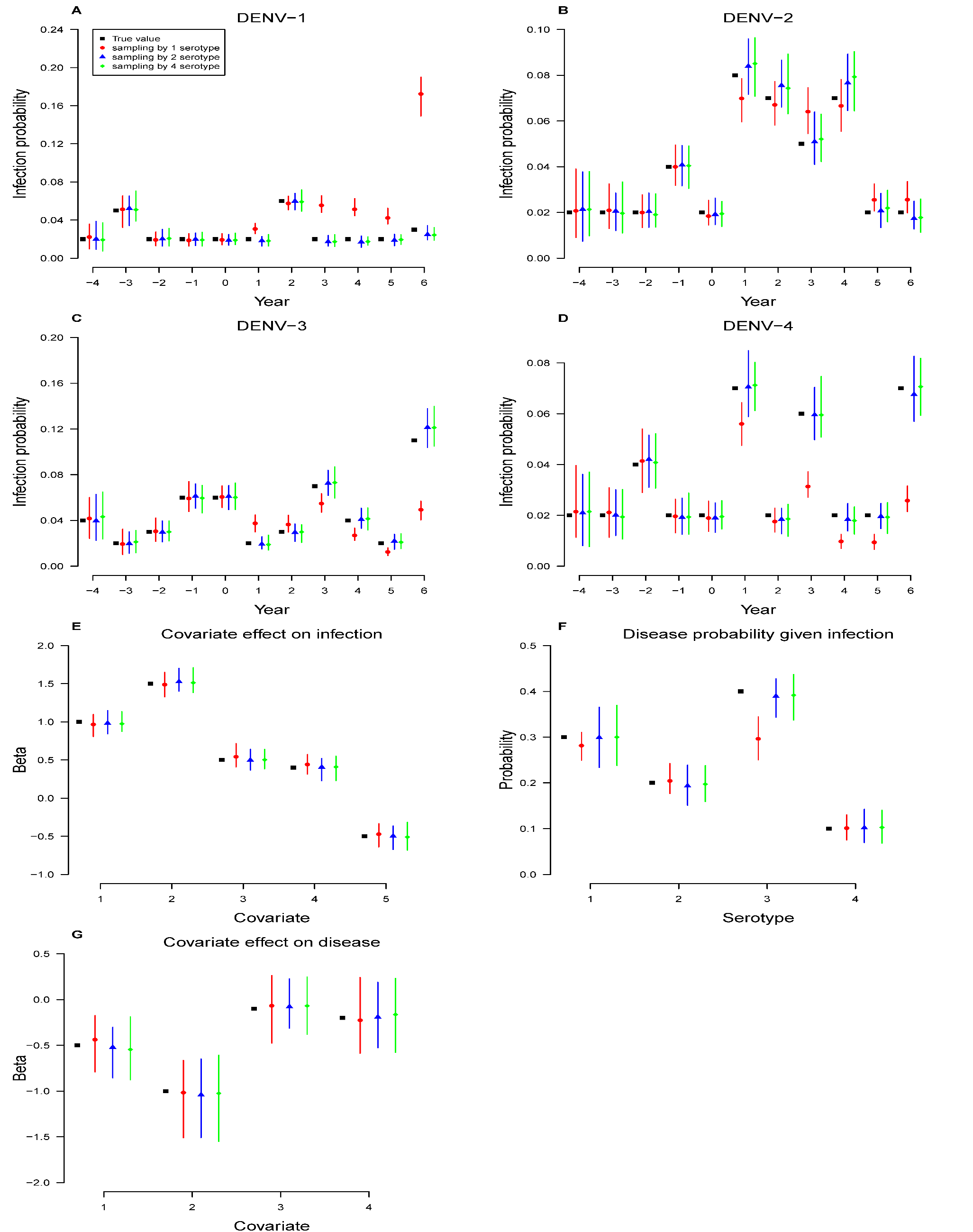
**

**Supplementary Figure 3.** Simulation study for comparing 1-serotype (red dot), 2-serotype (blue triangle) and 4-serotype (green diamond) sampling approaches for individual infection history. True values are denoted by black square points. Posterior medians (points) and the 2.5% and 97.5% quantiles (bars) of the estimates over 100 simulations are shown for annual infection probabilities for DENV1-DENV4 (panels A-D, respectively), covariate effects on probability of infection (panel E), probabilities of disease given infection for all serotypes (panel F), and covariate effects on probability of disease given infection (panel G). Covariates 1- 2 indicate the number of prior infections =1 and >1 respectively, 3-4 indicate =2 and >2 years since the most recent infection respectively, and 5 indicates age >8. Source data are provided as a Source Data file.


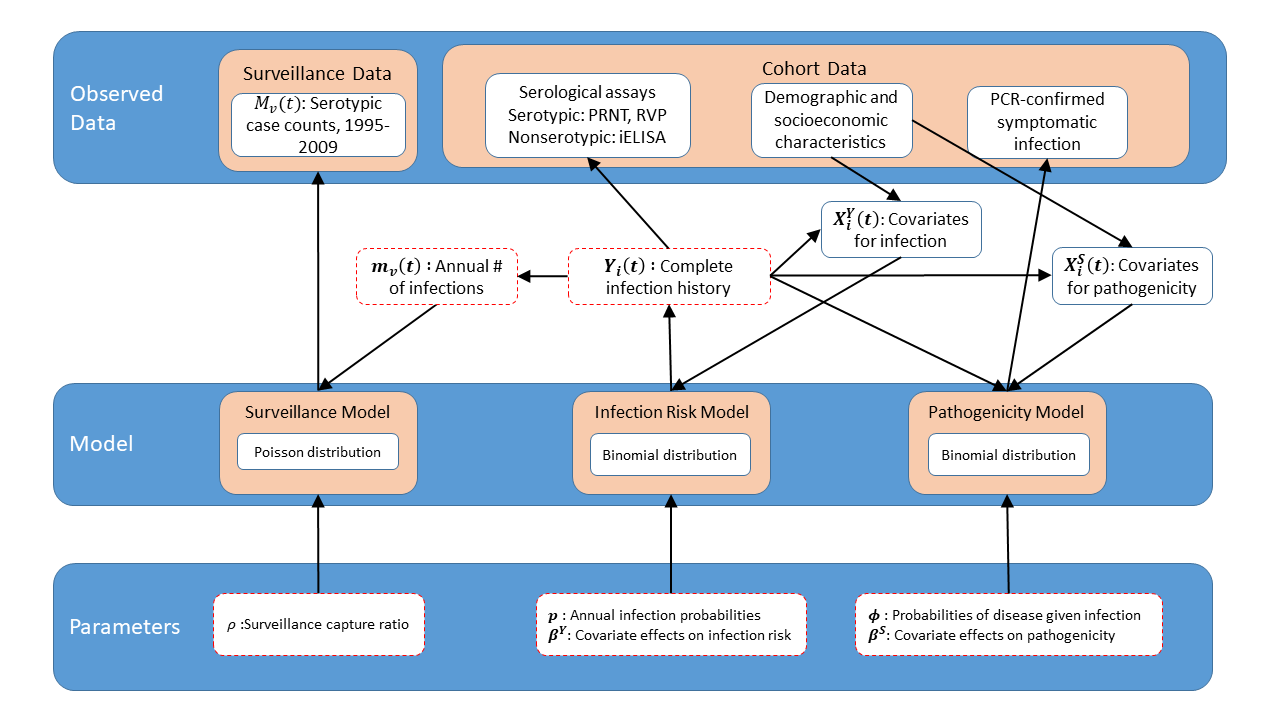


**Supplementary Figure 4.** Schematic of the hierarchical modeling framework based on data generation or dependence. Model components in the middle layer take parameters and risk factors (covariates) as inputs, and output infection histories, disease status, and surveillance data. Infection histories also serve as inputs for surveillance and pathogenicity models, and provide additional inputs for infection and pathogenicity models as covariates. Serological data are generated from infection histories by laboratory assays. The outcomes of the model are posterior samples of parameters and the infection histories, denoted by dashed boxes.

# SUPPLEMENTARY TABLES

**Supplementary Table 1**. Data summary by demographics, laboratory evaluation methods, laboratory test results and study year for the Nicaragua Pediatric Dengue Cohort. Column percentages are given in parentheses.

| Variable | Category | Epidemic Years | | | | | |
| --- | --- | --- | --- | --- | --- | --- | --- |
|  |  | 2004-2005 | 2005-2006 | 2006-2007 | 2007-2008 | 2008-2009 | 2009-2010 |
| # of subject |  | 3426 | 3446 | 3171 | 3337 | 3560 | 3601 |
| Age | 2-5 | 1404 (41) | 1074 (31) | 873 (28) | 846 (25) | 837 (24) | 698 (19) |
|  | 6-8 | 1284 (37) | 1283 (37) | 1178 (37) | 1111 (33) | 1018 (29) | 953 (26) |
|  | >8 | 738 (22) | 1089 (32) | 1120 (35) | 1380 (41) | 1705 (48) | 1950 (54) |
| Sex | Male | 1682 (49) | 1693 (49) | 1575 (50) | 1650 (49) | 1766 (50) | 1790 (50) |
|  | Female | 1744 (51) | 1753 (51) | 1596 (50) | 1687 (51) | 1794 (50) | 1811 (50) |
| # of lab-tested pairs of annual sera | NT | 87 (3) | 93 (3) | 97 (3) | 97 (3) | 104 (3) | 77 (2) |
|  | PRNT | 361 (11) | 357 (10) | 376 (12) | 0 (0) | 0 (0) | 0 (0) |
|  | iELISA | 2961 (86) | 2931 (85) | 2685 (85) | 3176 (95) | 3434 (96) | 3354 (93) |
| # of infections by lab-test method | RT-PCR^1^ | 17 (6) | 65 (16) | 13 (6) | 64 (26) | 22 (10) | 170 (41) |
|  | NT | 31 (11) | 34 (8) | 22 (10) | 27 (11) | 22 (10) | 16 (4) |
|  | PRNT | 29 (10) | 35 (9) | 34 (15) | 0 (0) | 0 (0) | 0 (0) |
|  | iELISA | 211 (73) | 271 (67) | 152 (69) | 159 (64) | 179 (80) | 228 (55) |
|  | Overall | 288 | 405 | 221 | 250 | 223 | 414 |
| # of infections by serotype^2^ | DENV-1 | 29 (10) | 42 (10) | 17 (8) | 9 (4) | 3 (1) | 22 (5) |
|  | DENV-2 | 31 (11) | 66 (16) | 26 (12) | 72 (29) | 8 (4) | 10 (2) |
|  | DENV-3 | 6 (2) | 10 (2) | 8 (4) | 6 (2) | 30 (13) | 149 (36) |
|  | DENV-4 | 7 (2) | 6 (1) | 15 (7) | 2 (1) | 2 (1) | 3 (1) |
|  | Unknown | 214 (74) | 280 (69) | 155 (70) | 161 (64) | 180 (81) | 230 (56) |

^1^Symptomatic dengue cases who were RT-PCR-negative but confirmed by iELISA on paired acute and convalescent sera are included here.

^2^2 patients, one in 2004-05 and the other in 2005-06, had co-infections of two serotypes by RT-PCR.

**Supplementary Table 2**. Serotype-specific infection numbers by laboratory test and demographics. Column percentages are given in parentheses.

| Variable | Category | Serotype | | | | |
| --- | --- | --- | --- | --- | --- | --- |
|  |  | DENV-1 | DENV-2 | DENV-3 | DENV-4 | Unknown |
| Laboratory Test | RT-PCR | 53 (43) | 115 (54) | 160 (77) | 1 (3) | 22 (2) |
|  | NT | 41 (34) | 58 (27) | 33 (16) | 20 (57) | 0 (0) |
|  | PRNT | 28 (23) | 40 (19) | 16 (8) | 14 (40) | 0 (0) |
|  | iELISA | 0 (0) | 0 (0) | 0 (0) | 0 (0) | 1200 (98) |
| Age | 2-5 | 43 (35) | 59 (28) | 41 (20) | 8 (23) | 330 (27) |
|  | 6-8 | 38 (31) | 69 (32) | 64 (31) | 14 (40) | 425 (35) |
|  | >8 | 41 (34) | 85 (40) | 104 (50) | 13 (37) | 467 (38) |
| Sex | Male | 52 (43) | 96 (45) | 103 (49) | 16 (46) | 593 (49) |
|  | Female | 70 (57) | 117 (55) | 106 (51) | 19 (54) | 629 (51) |
| Total |  | 122 | 213 | 209 | 35 | 1222 |

**Supplementary Table 3**. Model estimates (posterior median and 95% credible interval [CI]) for serotype-specific probabilities of infection from 1995 to 2009. The proportion of non-dominant serotypes (reported by virological surveillance) among all surveillance-reported cases is assumed to be 10% for the pre-study years.

|  | Serotype | | | |
| --- | --- | --- | --- | --- |
| Year | DENV-1 | DENV-2 | DENV-3 | DENV-4 |
| 1995-1996 | 0.047 (0.029, 0.07) | 0.048 (0.028, 0.073) | 0.265 (0.225, 0.312) | 0.013 (0.004, 0.029) |
| 1996-1997 | 0.001 (0, 0.003) | 0.001 (0, 0.003) | 0.071 (0.053, 0.093) | 0.001 (0, 0.003) |
| 1997-1998 | 0 (0, 0.002) | 0 (0, 0.002) | 0.079 (0.063, 0.098) | 0 (0, 0.002) |
| 1998-1999 | 0.005 (0.002, 0.01) | 0.033 (0.024, 0.042) | 0.272 (0.238, 0.312) | 0.005 (0.002, 0.01) |
| 1999-2000 | 0.005 (0.002, 0.009) | 0.231 (0.202, 0.265) | 0.033 (0.024, 0.042) | 0.005 (0.002, 0.009) |
| 2000-2001 | 0.002 (0.001, 0.005) | 0.146 (0.126, 0.168) | 0.017 (0.012, 0.025) | 0.012 (0.008, 0.018) |
| 2001-2002 | 0 (0, 0.002) | 0.043 (0.033, 0.054) | 0.002 (0.001, 0.005) | 0 (0, 0.002) |
| 2002-2003 | 0.051 (0.042, 0.062) | 0.005 (0.003, 0.009) | 0.001 (0, 0.003) | 0.005 (0.003, 0.009) |
| 2003-2004 | 0.109 (0.095, 0.124) | 0 (0, 0.001) | 0 (0, 0.001) | 0 (0, 0.001) |
| 2004-2005 | 0.063 (0.047, 0.074) | 0.021 (0.016, 0.031) | 0.002 (0.001, 0.006) | 0.003 (0.002, 0.019) |
| 2005-2006 | 0.037 (0.03, 0.044) | 0.08 (0.065, 0.091) | 0.004 (0.002, 0.014) | 0.003 (0.001, 0.012) |
| 2006-2007 | 0.009 (0.007, 0.013) | 0.054 (0.045, 0.063) | 0.002 (0.001, 0.005) | 0.007 (0.004, 0.011) |
| 2007-2008 | 0.004 (0.002, 0.01) | 0.075 (0.064, 0.086) | 0.004 (0.002, 0.006) | 0.001 (0, 0.002) |
| 2008-2009 | 0.004 (0.002, 0.007) | 0.005 (0.002, 0.008) | 0.05 (0.042, 0.059) | 0.002 (0.001, 0.005) |
| 2009-2010 | 0.02 (0.015, 0.033) | 0.01 (0.005, 0.023) | 0.085 (0.065, 0.099) | 0.002 (0.001, 0.006) |

**Supplementary Table 4**. Estimates (posterior median and 95% CI) of the association between covariates on the probability of infection. Scenarios 1, 2 and 3 correspond to the assumptions of 5%, 10% and 15%, respectively, as the proportion of non-dominant serotypes (reported by virological surveillance) among all surveillance-reported cases for the pre-study years (Supplementary methods, Section 1.1).

| Variable | Category | Scenario 1 | Scenario 2 | Scenario 3 |
| --- | --- | --- | --- | --- |
| Age | 2-8 | Ref | Ref | Ref |
|  | >8 | 1.54 (1.37, 1.73) | 1.55 (1.39, 1.74) | 1.55 (1.38, 1.74) |
| # of prior infection | 0 | Ref | Ref | Ref |
|  | 1 | 0.47 (0.35, 0.62) | 0.46 (0.34, 0.6) | 0.43 (0.33, 0.57) |
|  | >1 | 1.93 (1.29, 2.81) | 1.91 (1.34, 2.64) | 1.71 (1.22, 2.38) |
| # of prior infections & years since last infection | 1, 1 year | Ref | Ref | Ref |
|  | 1, 2 years | 1.00 (0.64, 1.56) | 1.12 (0.73, 1.69) | 1.22 (0.8, 1.79) |
|  | 1, >2 years | 1.46 (1.07, 2.04) | 1.47 (1.1, 2.06) | 1.55 (1.13, 2.02) |
|  | >1, 1 year | Ref | Ref | Ref |
|  | >1, 2 years | 0.77 (0.44, 1.3) | 0.76 (0.46, 1.27) | 0.8 (0.48, 1.29) |
|  | >1, >2 years | 0.65 (0.41, 1.04) | 0.66 (0.44, 1.02) | 0.75 (0.5, 1.14) |
| Home ownership | No | Ref | Ref | Ref |
|  | Yes | 0.79 (0.71, 0.9) | 0.81 (0.71, 0.93) | 0.82 (0.72, 0.94) |
| # of fans | 0 | Ref | Ref | Ref |
|  | 1-2 | 0.67 (0.6, 0.75) | 0.68 (0.59, 0.77) | 0.68 (0.6, 0.77) |
|  | 3-4 | 0.67 (0.58, 0.76) | 0.67 (0.58, 0.78) | 0.67 (0.59, 0.77) |
|  | 5-6 | 0.54 (0.44, 0.66) | 0.55 (0.45, 0.66) | 0.55 (0.45, 0.67) |
|  | >6 | 0.55 (0.39, 0.75) | 0.56 (0.4, 0.77) | 0.56 (0.4, 0.76) |

**Supplementary Table 5**. The Spearman correlation between socioeconomic variables and infection with any DENV during the study period. Levels of each variable are ranked by their socioeconomic implications, e.g., for school type, 1=public, 2=semi-private, and 3=private.

| Variable | Levels | # of infections | # of person-years | Incidence(×10^-2^) | Correlation | p-value |
| --- | --- | --- | --- | --- | --- | --- |
| School type | Public | 1092 | 12267 | 8.90 | -0.035 | 0.053 |
|  | Semi-Private | 64 | 888 | 7.21 |  |  |
|  | Private | 186 | 2354 | 7.90 |  |  |
| Father education | None | 518 | 5770 | 8.98 | -0.027 | 0.101 |
|  | Primary | 301 | 3309 | 9.10 |  |  |
|  | Secondary | 603 | 7355 | 8.20 |  |  |
|  | Technical | 11 | 82 | 13.41 |  |  |
|  | University | 66 | 784 | 8.42 |  |  |
| Mother education | None | 173 | 1951 | 8.87 | -0.03 | 0.067 |
|  | Primary | 468 | 5025 | 9.31 |  |  |
|  | Secondary | 806 | 9580 | 8.41 |  |  |
|  | Technical | 20 | 224 | 8.93 |  |  |
|  | University | 51 | 701 | 7.28 |  |  |
| Household size | 1-2 | 17 | 138 | 12.32 | -0.003 | 0.839 |
|  | 3-4 | 224 | 2728 | 8.21 |  |  |
|  | 5-6 | 446 | 4811 | 9.27 |  |  |
|  | >6 | 941 | 11144 | 8.44 |  |  |
| Position of faucet | No faucet | 13 | 121 | 10.74 | -0.004 | 0.824 |
|  | Outside | 504 | 5626 | 8.96 |  |  |
|  | Inside | 949 | 11048 | 8.59 |  |  |
|  | Both | 117 | 1393 | 8.40 |  |  |
| Faucet shared or not | No faucet | 11 | 141 | 7.80 | -0.016 | 0.346 |
|  | Shared | 173 | 1791 | 9.66 |  |  |
|  | Not shared | 1330 | 15505 | 8.58 |  |  |
| Floor type | Ground | 368 | 3793 | 9.70 | -0.008 | 0.636 |
|  | Bricks | 659 | 8165 | 8.07 |  |  |
|  | Concrete | 474 | 5214 | 9.09 |  |  |
|  | Ceramic tile | 82 | 1018 | 8.05 |  |  |
| Ceiling type | Roof tiles | 5 | 37 | 13.51 | -0.029 | 0.075 |
|  | Plastic | 4 | 29 | 13.79 |  |  |
|  | Zinc | 1564 | 18035 | 8.67 |  |  |
| Home ownership | No | 144 | 1391 | 10.35 | -0.04 | 0.013 |
|  | Yes | 1439 | 16799 | 8.57 |  |  |
| # of electric fans | 0 | 132 | 1425 | 9.26 | -0.033 | 0.041 |
|  | 1-2 | 968 | 10823 | 8.94 |  |  |
|  | 3-4 | 373 | 4518 | 8.26 |  |  |
|  | 5-6 | 91 | 1141 | 7.97 |  |  |
|  | >6 | 20 | 299 | 6.69 |  |  |
| # of television sets | 0 | 30 | 296 | 10.13 | -0.017 | 0.296 |
|  | 1-2 | 1229 | 14032 | 8.76 |  |  |
|  | 3-4 | 266 | 3260 | 8.16 |  |  |
|  | 5-6 | 45 | 480 | 9.38 |  |  |
|  | >6 | 14 | 138 | 10.14 |  |  |
| # of refrigerators | 0 | 538 | 6014 | 8.95 | -0.014 | 0.386 |
|  | 1-2 | 1021 | 11908 | 8.57 |  |  |
|  | 3-4 | 18 | 252 | 7.14 |  |  |
|  | 5-6 | 3 | 20 | 15 |  |  |
| Own motorcylce | No | 1473 | 16880 | 8.73 | -0.004 | 0.799 |
|  | Yes | 106 | 1297 | 8.17 |  |  |
| Own cars | No | 1408 | 16119 | 8.73 | -0.017 | 0.284 |
|  | Yes | 171 | 2058 | 8.31 |  |  |
| Chickens in house | No | 1401 | 16000 | 8.76 | -0.019 | 0.252 |
|  | Yes | 111 | 1417 | 7.83 |  |  |
| Ducks in house | No | 1477 | 17001 | 8.69 | 0.005 | 0.78 |
|  | Yes | 35 | 416 | 8.41 |  |  |
| Dogs in house | No | 976 | 11495 | 8.49 | 0.023 | 0.167 |
|  | Yes | 536 | 5922 | 9.05 |  |  |
| Cats in house | No | 1319 | 15262 | 8.64 | 0.006 | 0.72 |
|  | Yes | 194 | 2167 | 8.95 |  |  |
| Birds in house | No | 1446 | 16589 | 8.72 | -0.015 | 0.366 |
|  | Yes | 67 | 840 | 7.98 |  |  |
| Pigs in house | No | 1506 | 17372 | 8.67 | 0.012 | 0.464 |
|  | Yes | 7 | 57 | 12.28 |  |  |
| Rats in house | No | 201 | 2395 | 8.39 | 0.012 | 0.47 |
|  | Yes | 1312 | 15034 | 8.73 |  |  |

**Supplementary Table 6**. Estimates (posterior median and 95% CI) of the probability of symptoms given infection and the associated effects of covariates. Scenarios 1, 2 and 3 correspond to the assumptions of 5%, 10% and 15%, respectively, as the proportion of non-dominant serotypes (reported by virological surveillance) among all surveillance-reported cases for the pre-study years (Supplementary methods, Section 1.1).

| Parameter | Variable | Category | Scenario 1 | Scenario 2 | Scenario 3 |
| --- | --- | --- | --- | --- | --- |
| Probability of symptoms |  | DENV-1 | 0.10 (0.08, 0.14) | 0.10 (0.07, 0.13) | 0.09 (0.07, 0.12) |
|  |  | DENV-2 | 0.13 (0.11, 0.16) | 0.13 (0.11, 0.16) | 0.14 (0.11, 0.18) |
|  |  | DENV-3 | 0.25 (0.2, 0.31) | 0.24 (0.19, 0.3) | 0.26 (0.21, 0.31) |
|  |  | DENV-4 | 0.02 (0, 0.06) | 0.02 (0, 0.06) | 0.01 (0, 0.05) |
| Odds ratio | Age | 2-8 | Ref | Ref | Ref |
|  |  | >8 | 2.11 (1.4, 3.15) | 2.2 (1.46, 3.37) | 2.16 (1.41, 3.3) |
|  | Age & years since last infection | 2-8, 1 year | Ref | Ref | Ref |
|  |  | 2-8, 2 years | 1.71 (0.7, 4.09) | 1.57 (0.6, 3.83) | 1.54 (0.64, 3.78) |
|  |  | 2-8, >2 years | 0.89 (0.42, 1.93) | 0.92 (0.41, 2.05) | 0.90 (0.44, 2.16) |
|  |  | >8, 1 year | Ref | Ref | Ref |
|  |  | >8, 2 years | 0.19 (0.04, 0.78) | 0.19 (0.03, 0.77) | 0.18 (0.03, 0.78) |
|  |  | >8, >2 years | 0.73 (0.34, 1.75) | 0.71 (0.33, 1.63) | 0.67 (0.29, 1.6) |
|  | Age & # of prior infection | 2-8, 0 | Ref | Ref | Ref |
|  |  | 2-8, 1 | 0.81 (0.38, 1.58) | 0.82 (0.39, 1.68) | 0.83 (0.35, 1.61) |
|  |  | 2-8, >1 | 1.19 (0.44, 2.74) | 1.31 (0.49, 3.11) | 1.34 (0.51, 3.16) |
|  |  | >8, 0 | Ref | Ref | Ref |
|  |  | >8, 1 | 1.03 (0.39, 2.35) | 1.07 (0.43, 2.52) | 1.15 (0.46, 3.09) |
|  |  | >8, >1 | 1.27 (0.51, 2.86) | 1.36 (0.55, 3.07) | 1.41 (0.6, 3.45) |

**Supplementary Table 7**. Estimated effects (posterior median and 95% CI) of iELISA levels on the probability of secondary infection, probability of symptomatic secondary infection, and probability of disease given secondary infection among children with one or more prior infections. Unobserved infection outcomes are sampled from their posterior distributions. Only the data during the study period are used as antibody levels are not available before the study.

| Outcome | Secondary infection | Symptomatic Secondary Infection | Disease given  secondary infection |
| --- | --- | --- | --- |
| Undetectable | Ref | Ref | Ref |
| ≤20 | 3.13 (2.16, 4.52) | 2.18 (0.8, 5.94) | 1.20 (0.39, 3.77) |
| 21-80 | 1.83 (1.28, 2.62) | 2.30 (0.87, 6.05) | 1.29 (0.43, 3.91) |
| 81-320 | 1.11 (0.77, 1.59) | 1.19 (0.45, 3.17) | 1.01 (0.33, 3.1) |
| 321-1280 | 0.72 (0.49, 1.07) | 1.13 (0.41, 3.11) | 1.59 (0.49, 5.17) |
| >1280 | 0.38 (0.24, 0.6) | 1.17 (0.4, 3.46) | 3.60 (0.99, 13.09) |

**Supplementary Table 8**. Sensitivity analysis for estimating the effects of iELISA levels on the probability of secondary infection, probability of symptomatic secondary infection, and probability of disease given secondary infection among children with one or more prior infections. For person-years with a preseason iELISA titer of 1280 or higher, the definition of infection is relaxed to either seroconversion or a ≥ 2-fold increase between paired annual sera. Other settings remain the same as Supplementary Table 9.

| Outcome | Secondary infection | symptomatic secondary infection | Disease given  secondary infection |
| --- | --- | --- | --- |
| Undetectable | Ref | Ref | Ref |
| ≤20 | 3.12 (2.16, 4.5) | 2.18 (0.8, 5.93) | 1.23 (0.4, 3.82) |
| 21-80 | 1.82 (1.27, 2.61) | 2.30 (0.88, 6.03) | 1.32 (0.44, 3.97) |
| 81-320 | 1.10 (0.77, 1.58) | 1.19 (0.45, 3.16) | 1.03 (0.34, 3.15) |
| 321-1280 | 0.72 (0.49, 1.06) | 1.13 (0.41, 3.1) | 1.62 (0.5, 5.21) |
| >1280 | 0.90 (0.59, 1.37) | 1.17 (0.39, 3.5) | 1.09 (0.31, 3.87) |

**Supplementary Table 9**. Assessing goodness-of-fit: observed and model-predicted annual attack rates during the study period, where model prediction is based on simulated epidemics using posterior samples of parameters.

| Study year | Annual Attack rate | | | |
| --- | --- | --- | --- | --- |
|  | Observed | Model-predicted quantiles | | |
|  |  | Median (50%) | 2.5% | 97.5% |
| 2004 | 8.41% | 8.58% | 7.30% | 9.95% |
| 2005 | 11.71% | 10.67% | 9.28% | 12.11% |
| 2006 | 6.90% | 5.83% | 4.80% | 6.99% |
| 2007 | 7.41% | 6.52% | 5.43% | 7.74% |
| 2008 | 6.24% | 6.02% | 4.98% | 7.16% |
| 2009 | 11.50% | 11.52% | 10.08% | 13.05% |

**Supplementary Table 10.** Assessing goodness-of-fit: observed and model-predicted annual numbers of dengue infections, with and without stratification by age group. The model prediction in each study year is conditional on observed infection history in the past.

|  | Overall | | Stratified by Age Group | | |
| --- | --- | --- | --- | --- | --- |
| Study year | Observed | Model-predicted | Age Group | Observed | Model-predicted |
| 2004 | 322 | 324 | 2-8 y | 262 | 258 |
|  |  |  | >8 y | 60 | 66 |
| 2005 | 442 | 445 | 2-8 y | 308 | 322 |
|  |  |  | >8 y | 134 | 122 |
| 2006 | 238 | 240 | 2-8 y | 163 | 169 |
|  |  |  | >8 y | 74 | 71 |
| 2007 | 267 | 271 | 2-8 y | 174 | 180 |
|  |  |  | >8 y | 94 | 91 |
| 2008 | 229 | 232 | 2-8 y | 102 | 123 |
|  |  |  | >8 y | 127 | 109 |
| 2009 | 414 | 416 | 2-8 y | 192 | 183 |
|  |  |  | >8 y | 222 | 233 |
| *P*-value^1^ | 0.997 | | 0.363 | | |

^1^ P-value is based on the Chi-squared test for testing goodness-of-fit for logistic regression.

**Supplementary Table 11**. Estimates (posterior median and 95% CI) of the probability of disease given infection and the associated effects of covariates. Different from Supplementary Table 6, the probability of developing symptoms given infection is stratified by the number of prior infections (0 vs. ≥1) for DENV-1, DENV-2 and DENV-3. This stratification is not done for DENV-4 as there was only one PCR-confirmed symptomatic infection with DENV-4. Only scenario 2 is presented, i.e., 10% is assumed as the proportion of non-dominant serotypes (reported by virological surveillance) among all surveillance-reported cases for the pre-study years.

| Parameter | Variable | Category | Scenario 2 |
| --- | --- | --- | --- |
| Probability of symptoms |  | DENV-1 | 0.13 (0.09, 0.19) |
|  |  | DENV-2 | 0.1 (0.07, 0.13) |
|  |  | DENV-3 | 0.27 (0.2, 0.39) |
|  |  | DENV-4 | 0.02 (0, 0.06) |
| Odds ratio | Age | 2-8 | Ref |
|  |  | >8 | 2.09 (1.4, 3.14) |
|  | Age & years since last infection | 2-8, 1 year | Ref |
|  |  | 2-8, 2 years | 1.23 (0.58, 3.04) |
|  |  | 2-8, >2 years | 0.79 (0.43, 1.53) |
|  |  | >8, 1 year | Ref |
|  |  | >8, 2 years | 0.24 (0.05, 0.89) |
|  |  | >8, >2 years | 0.84 (0.48, 1.69) |
|  | # of prior infection (Ref=0) | ≥1, serotype 1 | 0.57 (0.24, 1.14) |
|  |  | ≥1, serotype 2 | 1.99 (1.02, 3.62) |
|  |  | ≥1, serotype 3 | 0.71 (0.31, 1.4) |

**Supplementary Table 12**. Crude probabilities of disease given infection by demographics and infection history.

| Variable | Category | All | Baseline Serostatus | |
| --- | --- | --- | --- | --- |
|  |  |  | Seronegative | Seropositive^1^ |
| # of person-years |  | 1801 | 868 | 933 |
| Age | 2-8 | 171/1091 (15.7%) | 122/667 (18.3%) | 49/424 (11.6%) |
|  | >8 | 180/710 (25.4%) | 59/201 (29.4%) | 121/509 (23.8%) |
| Sex | Male | 171/860 (19.9%) | 88/418 (21.1%) | 83/442 (18.8%) |
|  | Female | 180/941 (19.1%) | 93/450 (20.7%) | 87/491 (17.7%) |
| # of prior infections | 0 | 148/719 (20.6%) | 148/719 (20.6%) | NA |
|  | 1 | 166/949 (17.5%) | 27/125 (21.6%) | 139/824 (16.9%) |
|  | >1 | 37/133 (27.8%) | 6/24 (25%) | 31/109 (28.4%) |
| Years since last infection | 1 | 22/103 (21.4%) | 12/71 (16.9%) | 10/32 (31.2%) |
|  | 2 | 11/64 (17.2%) | 8/40 (20%) | 3/24 (12.5%) |
|  | >2 | 31/91 (34.1%) | 13/38 (34.2%) | 18/53 (34%) |
| Age & years since last infection | 2-8, 1 year | 11/67 (16.4%) | 7/53 (13.2%) | 4/14 (28.6%) |
|  | 2-8, 2 years | 8/34 (23.5%) | 7/24 (29.2%) | 1/10 (10%) |
|  | 2-8, >2 years | 8/23 (34.8%) | 6/18 (33.3%) | 2/5 (40%) |
|  | >8, 1 year | 11/36 (30.6%) | 5/18 (27.8%) | 6/18 (33.3%) |
|  | >8, 2 years | 3/30 (10%) | 1/16 (6.2%) | 2/14 (14.3%) |
|  | >8, >2 years | 23/68 (33.8%) | 7/20 (35%) | 16/48 (33.3%) |
| Age & # of prior infection | 2-8, 0 | 102/572 (17.8%) | 102/572 (17.8%) | NA |
|  | 2-8, 1 | 60/479 (12.5%) | 18/84 (21.4%) | 42/395 (10.6%) |
|  | 2-8, >1 | 9/40 (22.5%) | 2/11 (18.2%) | 7/29 (24.1%) |
|  | >8, 0 | 46/147 (31.3%) | 46/147 (31.3%) | NA |
|  | >8, 1 | 106/470 (22.6%) | 9/41 (22%) | 97/429 (22.6%) |
|  | >8, >1 | 28/93 (30.1%) | 4/13 (30.8%) | 24/80 (30%) |

^1^Assuming all baseline-positive individuals had exactly one prior infection before the study entry.

**Supplementary Table 13**. Estimates (posterior median and 95% CI) of the probability of symptoms given infection and the associated effects of covariates. Different from Supplementary Table 6, the effect of years since last infection is stratified by the number of prior infections rather than age group. Only scenario 2 is presented, i.e., 10% is assumed as the proportion of non-dominant serotypes (reported by virological surveillance) among all surveillance-reported cases for the pre-study years.

| Parameter | Variable | Category | Scenario 2 |
| --- | --- | --- | --- |
| Probability of symptoms |  | DENV-1 | 0.10 (0.07, 0.13) |
|  |  | DENV-2 | 0.14 (0.11, 0.17) |
|  |  | DENV-3 | 0.24 (0.19, 0.30) |
|  |  | DENV-4 | 0.02 (0, 0.06) |
| Odds ratio | Age | 2-8 | Ref |
|  |  | >8 | 1.98 (1.46, 2.62) |
|  | Number of prior infection | 0 | Ref |
|  |  | 1 | 0.75 (0.32, 1.5) |
|  |  | >1 | 1.97 (0.86, 4.11) |
|  | Number of prior & years since last infection | 1, 1 year | Ref |
|  |  | 1, 2 years | 1.49 (0.56, 3.82) |
|  |  | 1, >2 years | 1.05 (0.51, 2.21) |
|  |  | >1, 1 year | Ref |
|  |  | >1, 2 years | 0.19 (0.04, 0.76) |
|  |  | >1, >2 years | 0.53 (0.22, 1.3) |
